# Analysis of Japanese Articles about Suicides Involving Charcoal Burning or Hydrogen Sulfide Gas

**DOI:** 10.3390/ijerph13101013

**Published:** 2016-10-15

**Authors:** Yoshihiro Nabeshima, Daisuke Onozuka, Takanari Kitazono, Akihito Hagihara

**Affiliations:** 1 Department of Medicine and Clinical Science, Graduate School of Medical Sciences, Kyushu University, 3-1-1 Maidashi, Higashi-ku, Fukuoka 812-8582, Japan; kendai.nabe@gmail.com (Y.N.); kitazono@intmed2.med.kyushu-u.ac.jp (T.K.); 2 Department of Health Communication, Graduate School of Medical Sciences, Kyushu University, 3-1-1 Maidashi, Higashi-ku, Fukuoka 812-8582, Japan; onozukad@hcam.med.kyushu-u.ac.jp

**Keywords:** charcoal, hydrogen sulfide, suicide, newspaper article, report, World Health Organization (WHO), guideline

## Abstract

It is well known that certain types of media reports about suicide can result in imitative suicides. In the last two decades, Japan has experienced two suicide epidemics and the subsequent excessive media coverage of these events. However, the quality of the media suicide reports has yet to be evaluated in terms of the guidelines for media suicide coverage. Thus, the present study analyzed Japanese newspaper articles (*n* = 4007) on suicides by charcoal burning or hydrogen sulfide gas between 11 February 2003 and 13 March 2010. The suicide reports were evaluated in terms of the extent to which they conformed to the suicide reporting guidelines. The mean violation scores were 3.06 (±0.7) for all articles, 3.2 (±0.8) for articles about suicide by charcoal burning, and 2.9 (±0.7) for articles about suicide by hydrogen sulfide (*p* < 0.001). With the exception of not following several recommendations, newspaper articles about suicide have improved in quality, as defined by the recommendations for media suicide coverage. To prevent imitative suicides based on media suicide reports, individuals in the media should try not to report suicide methods and to make attempts to report the poor condition of suicide survivors.

## 1. Introduction

Following the publication of a book entitled “The Sorrows of Young Werther” by Goethe in 1774, many young people who were affected by the book tried to commit suicide; this phenomenon is often referred to as the Werther effect [1,2,3,4]. It has been suggested that media suicide reports might influence viewers and readers with suicidal ideation and result in an increased number of suggested suicides. As a result, the potential association between the media coverage of a suicide and subsequent actual suicidality (i.e., fatal and non-fatal suicidal acts or suicidal ideation) has been extensively investigated [4,5,6,7,8,9,10,11]. A systematic review of previous studies suggests that the media impact suicide rates [1,5,6,12]. More specifically, the contagion effect associated with media reports is higher when the number of stories about individual suicides increases and when media depictions of suicides include details about the suicide and the method used. Additionally, the contagion effect becomes even stronger if a particular death is reported for a long period of time, if an article about an individual death is placed on the front page, if there is more publicity, and if the case portrayed is a true story rather than fiction [13,14]. On the other hand, recent studies have suggested that certain types of suicide reports are associated with a decrease in the suicide rate; this effect is often referred as the Papageno effect [4]. Thus, based on these findings, a variety of recommendations for the media coverage of suicide has been proposed [3,15,16].

In the last two decades, Japan has experienced two different types of suicide epidemics and subsequent media reports. On 11 February 2003, a group of young people who met for the first time in Iruma City in the Saitama prefecture committed group suicide by carbon monoxide poisoning produced by burning charcoal. In the previous few days, the first three individuals who committed suicide had initially met through an Internet suicide site. This type of group suicide was unprecedented and peculiar and, thus, was repeatedly reported by the mass media in the ensuing days. Following the intensive reporting of this unusual case, group suicides by Japanese young people who met via the Internet and tried to commit suicide by burning charcoal drastically increased; this type of group suicide has been called net-jisatsu, or Internet suicide [17,18,19]. Another example of this phenomenon in Japan was suicide by inhaling hydrogen sulfide gas, which occurred in 2008. After the formula for making hydrogen sulfide at home was made publicly available online on 11 March 2008, the number of suicides in Japan by hydrogen sulfide exceeded 1000 individuals between January and November 2008, which was 35 times the number reported for a comparable period in 2007 [10,11,20,21]. This dramatic increase was presumed to be due to information about this suicide method available on websites [22].

Suicide attempts following the reporting of a suicide case by the mass media are often referred to as suggested suicides. Thus far, the Japanese government has been hesitant to develop an official version of the suicide event report guidelines or regulations out of consideration for freedom of the press. The Japanese newspapers are also supportive of the government attitude towards the media. Although guidelines for the reporting of suicides by Japanese media outlets in particular have yet to be published, suggested suicides associated with media reports are well-recognized based on the guidelines for mass media suicide coverage issued by the World Health Organization (WHO) and the Centers for Disease Control and Prevention [3,15,16]. Furthermore, in an effort to prevent suicide, appeals for emergency recommendations for suicide reporting by the media have been made by non-profit organizations (NPOs) [23], the Cabinet Office of the Japanese Government [24], and the Japanese Association for Suicide Prevention [25] during the last two decades. Several studies from Hong Kong, Korea, and Taiwan have investigated the quality of media suicide reports [26,27,28]. Several studies are specific to suicide in Japan [10,19]. However, to date, no studies have systematically analyzed the contents of the suicide-related articles published in national or local papers in Japan. As a result, it remains unclear whether the quality of newspaper articles about suicide have improved in terms of the definitions of the guidelines for mass media suicide coverage [3,15,16].

Therefore, the present study evaluated newspaper articles on those cases of suicide by charcoal burning that received intensive media coverage after 11 February 2003 as well as articles on those cases of suicide by hydrogen sulfide that received intensive media coverage after 11 March 2008 [29]. More specifically, this study: (1) examined the extent to which newspaper articles have followed the media guidelines for suicide reporting; (2) attempted to identify items in the suicide reporting guidelines that were likely to be either followed or ignored in reports of suicide cases; and (3) aimed to determine if the quality of newspaper articles on suicide that were published following the establishment of suicide report guidelines reflected improvement compared with those published before the guidelines. 

## 2. Methods

### 2.1. Newspaper Suicide Reports

Using the *Nikkei Telecom 21* newspaper database, the present study obtained all reports on suicides by charcoal burning or hydrogen sulfide that were published between February 2003 and March 2010 in the five largest-circulation Japanese newspapers (national papers) and in 39 local newspapers. There are five national papers that are distributed across Japan (2011 circulation figures in parentheses), *Yomiuri* (9,958,293), *Asahi* (11,075,893), *Mainichi* (4,545,855), *Nikkei* (4,620,928), and *Sankei* (2,313,297). The 39 local papers are based in each of the 47 prefectures in Japan. As some local papers cover multiple prefectures, the number of local papers was less than the number of prefectures. Thus, the present analyses covered all major newspapers in this country. 

The retrieval of relevant articles from the database was limited to those identified using the indexed search terms “*jisatsu*” (suicide) + “*rentan*” (charcoal) and “*jisatsu*” (suicide) + “*ryuka-suiso*” (hydrogen sulfide), and the search period was fixed between 11 February 2003 and 13 March 2010. Suicide using burning charcoal was first reported on 11 February 2003, and suicide using hydrogen sulfide was reported on 11 March 2008. Although suicide using burning charcoal has occurred since 11 March 2008, the incidence was not reported in the media because of the emergence of a new way for committing suicide using hydrogen sulfide. Thus, the charcoal burning suicide reports after 11 March 2008, were not analyzed in the study. After sales of chemical products that can be used to produce hydrogen sulfide gas were regulated [30], and an emergency appeal by the suicide prevention association was made [25], the numbers of suicides using hydrogen sulfide and subsequent newspaper reports decreased. In addition, a similar number of newspaper articles about suicide using hydrogen sulfide have accumulated (i.e., 1888 hydrogen sulfide vs. 2119 charcoal burning). Thus, we chose to analyze articles until March 2010. Of the 5246 articles that met the retrieval criteria, 4007 that reported attempted suicide cases were analyzed. Other types of articles (*n* = 1239), such as suicide-related reports of a court trial, suicide-related essays, or the introduction of a white paper on suicide, were excluded from the final analyses.

### 2.2. Database

For the present study, the content of each article analyzed was summarized, and a database was created based on the following variables: publication date (year, month, day of week); newspaper company; word count; page (front page or not); and whether information about the method of suicide, the existence of a suicide note, the reason for the suicide, where to seek help, the physical condition of the survivor after a suicide attempt, and the person who committed suicide (age, sex, occupation, address, and site of suicide) was included. Two raters coded the newspaper articles. If the raters had different opinions, they reached a consensus after a discussion, and resolved the conflict.

### 2.3. Data Analysis

The media suicide reporting guidelines of the WHO and other organizations consistently recommend the following when reporting a suicide case [3,15,16]: that the media avoid reporting a suicide on the front page and avoid mentioning the method of suicide, information about any suicide note, and the reason for the suicide. However, information about where depressed individuals can seek help and detailed information about the condition of an individual after a suicide attempt should be included in a suicide report. These items were used to calculate a suicide reporting violation score for each article as follows: (1) if an article was on the front page; (2) if an article reported the method of suicide; (3) if an article reported information about a suicide note left by the individual; (4) if an article reported the reason for the suicide; (5) if an article reported information about where depressed persons could seek help; and (6) if an article reported the condition of the person who attempted a suicide. Specifically, glorifying suicide by media report may affect readers [4]. The sixth item was recommended by the WHO when reporting suicide to avoid possible glorification [31]. Each article was assessed to determine the presence (Nos. 1–4) or absence (Nos. 5–6) of these items, and the total number of violations was calculated as the violation score; this score ranged from 0 to 6, and lower scores were indicative of fewer violations.

The study variables, including the violation score, were compared by type of suicide (i.e., suicide by charcoal burning or by hydrogen sulfide), and the violation score was used to sort the articles into three categories (0–2, 3–4, and 5–6) to identify recommendations that were more likely to be violated. Next, the proportion of violations with respect to the recommendations in each of the three categories was compared by type of suicide. Finally, to verify whether the quality of newspaper articles about suicide had improved with respect to observance of the media suicide report guidelines, four logistic regression models with type of suicide and various specific endpoints (Table 1) that served as dependent variables were fitted. Continuous variables were analyzed with *t*-tests, and categorical variables were analyzed with Chi-square (χ^2^) tests or Fisher’s exact tests. All data analyses were performed using JMP Pro ver.11.0 (SAS Institute Inc., Cary, NC, USA) for Mac, and *p*-values < 0.05 were considered to indicate statistical significance.

## 3. Results

**Figure 1:**
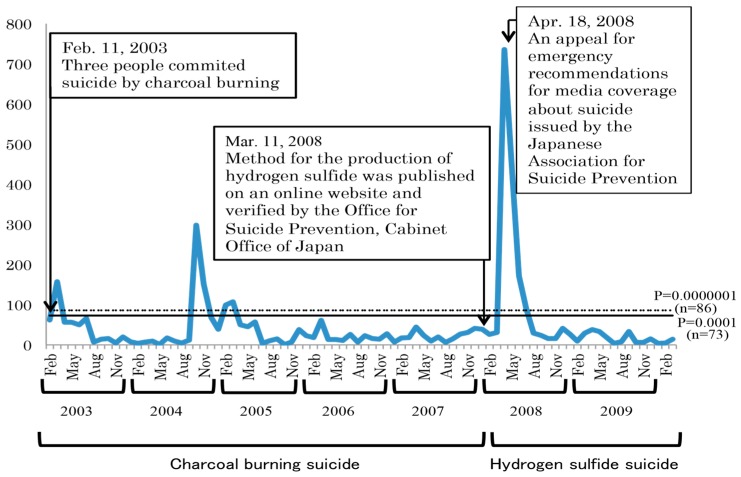
Number of newspaper articles about suicide by charcoal burning or hydrogen sulfide in Japan from 2003 to 2010.

The mean number of newspaper articles about suicide by charcoal burning or by hydrogen sulfide from 11 February 2003 to 13 March 2010 was 46.59 per month, and those that were significantly higher than expected according to the Poisson distribution are provided (*n* = 73, *p* = 0.0001; *n* = 87, *p* = 0.000001; Figure 1). The monthly number of newspaper articles about suicide increased rapidly after a suicide case using charcoal burning was first reported on 11 February 2003 and after the official verification of the hydrogen sulfide production method was posted on an online website on 11 March 2008.

The variables used in the present analyses are shown in Table 1 according to the type of suicide. During the study period, there were 2119 and 1888 newspaper articles that reported on suicides by charcoal burning and hydrogen sulfide, respectively, in Japan. With respect to the proportion of articles by month, there was a significant difference between the charcoal-burning and hydrogen-sulfide groups (*p* < 0.001) such that approximately 70% of articles about charcoal-burning suicides were reported in the January–March period, whereas 77% of the articles about the hydrogen-sulfide suicides were reported in the April–June period. The mean word count of the suicide-related articles was significantly lower in the hydrogen-sulfide group than in the charcoal-burning group (*p* < 0.001). In articles with a violation score of 6, a higher proportion of articles about suicide by hydrogen sulfide than about suicide by charcoal burning followed the recommendations; the exceptions concerned reporting the suicide method and not reporting the poor condition of the suicide survivor (all *p*-values < 0.001). 

Table 2 depicts the prevalence of newspaper articles about suicide by violation score and type of suicide. There was a significant difference in the proportion of newspaper articles about suicide (*p* < 0.001), and the proportion of articles in the 0–2 violation score group for hydrogen sulfide articles increased by 10.4% compared to the charcoal burning articles.

Table 3 shows the proportion of newspaper articles about suicide according to the type of suicide method and violation score. In the 0–2 group, higher proportions of articles violating recommendations 2 and 6 reported on suicide by hydrogen sulfide than on suicide by charcoal burning (*p* < 0.001). In the 3–4 group, lower proportions of articles violating recommendations 1, 3, 4, and 5 reported on suicide by hydrogen sulfide than on suicide by charcoal burning (all *p-*values < 0.001), whereas higher proportions of articles violating recommendations 2 and 6 reported on suicide by hydrogen sulfide than on suicide by charcoal burning (all *p-*values < 0.001). 

Table 4 presents the results of the logistic regression analyses for hydrogen-sulfide suicides (vs. charcoal-burning suicides) with respect to the six items that constitute the core of the recommendations. The newspaper articles about suicide by charcoal burning were published from 11 February 2003 to 10 March 2008, and the articles about suicide by hydrogen sulfide were published from 11 March 2008 to 13 March 2010. It has been reported that the word count of a suicide article, the newspaper company, month, and the day of the week of the article are related to the content of a newspaper suicide report [10,19,27,32]. Thus, the effects of these variables were controlled in the regression models. Since the periods of media coverage for the two types of suicide did not overlap, the type of suicide (charcoal burning vs. hydrogen sulfide) was used as an index of the period. Thus, the reporting year of an article was not controlled in the analysis.

The Hosmer-Lemeshow test indicated good fits for all the models, except the model that treated reporting information about where to seek help as a dependent variable (all *p-*values > 0.05). The four logistic regression analyses revealed that articles on suicide by hydrogen sulfide were less likely than articles on suicide by charcoal burning to violate the recommendations for media suicide coverage, except with regard to reporting the suicide method and not reporting the poor condition of the suicide survivor (all *p-*values < 0.001).

Following the official verification of the hydrogen sulfide production method on an online website, the number of articles about suicide by hydrogen sulfide rapidly increased (Figure 1). As a result, the Japanese Association for Suicide Prevention released an emergency appeal for recommendations for suicide reporting on 18 April 2008 [25]. To determine how the appeal for emergency recommendations influenced the quality of newspaper suicide-related articles, the proportions of newspaper articles about suicide that violated the WHO and other organizations’ recommendations for media suicide coverage were compared according to period of publication (i.e., pre-appeal: 11 March 2008–17 April 2008 vs. post-appeal: 18 April 2008–13 March 2010; Table 5). The proportions of articles that included information about a suicide note and the victim’s address significantly decreased after the emergency appeal (*p* = 0.006 and *p* = 0.004, respectively), but the proportion of articles that reported the reason for suicide significantly increased after the emergency appeal (*p* = 0.013).

## 4. Discussion

The present study comprehensively evaluated newspaper articles about suicide by charcoal burning or hydrogen sulfide between 11 February 2003 and 13 March 2010 and identified several new findings. First, the present findings revealed the extent to which national and local papers have followed the media suicide reporting guidelines. The mean violation scores were 3.06 (±0.7) for all articles, 3.2 (±0.8) for articles about suicide by charcoal burning, and 2.9 (±0.7) for articles about suicide by hydrogen sulfide. There was a significant difference between the violation scores of the articles about suicide by hydrogen sulfide and the articles about suicide by charcoal burning (*p* < 0.001; Table 1). The significant decrease in the violation score for newspaper articles about the two types of suicide may have been due to an increase in the number of articles about suicide by hydrogen sulfide in the 0–2 group (Table 2). Two different types of suicide epidemic have occurred in the last two decades in Japan. Because these types of suicide were unprecedented and relatively peculiar, mass media outlets repeatedly reported the cases and, following this intensive reporting, suggested suicide cases occurred [18,23,24,25]. As a result, the negative effects of inappropriate media coverage on suicides have become widely recognized; thus, the significant decreases in the violation scores may be due the widespread recognition of this issue by media institutions. 

The present study also identified the recommendations in the suicide reporting guidelines that were more likely to be followed and ignored in media reports. Of the six recommendations for suicide media coverage, those that were very likely to be violated included not reporting the suicide method (96.7%), not reporting information about where to seek help (88.6%), and not reporting the poor condition of the suicide survivor (89.3%; Table 1). Conversely, the recommendations that were likely to be followed included not publishing an article about a suicide on the front page (2.8%) and not reporting the reason for a suicide (7.8%). When analyzed by violation score, the proportions of newspaper articles that violated the media suicide report recommendations regarding not reporting about a suicide note and not reporting the reason for the suicide increased as the level of violated recommendations increased (Table 3). Additionally, the proportions of newspaper articles that violated the recommendations regarding not reporting the suicide method and reporting the poor condition of the suicide survivor were consistently high for all three violation-score groups. As mentioned above, because the suicide cases investigated in the present study were so unprecedented and so unusual, it may have been difficult for the mass media not to report the methods used in these cases [18,33]. However, as a practical implication of the present findings, it may be necessary to educate media professionals about not reporting suicide methods and reporting the poor condition of suicide survivors to prevent suggested suicides. 

Third, the present study aimed to determine whether the quality of newspaper articles about suicide had improved in terms of the media suicide reporting guidelines. In general, the quality of newspaper articles about suicide improved, but there were several exceptions; the exceptional items included not reporting the methods of suicide and reporting the poor condition of the suicide survivor. Again, because the suicide cases assessed in this study were so unprecedented and so unusual, it might have been difficult for mass media not to report the methods used [18,33]. During the publication periods (from 11 February 2003 to 10 March 2008 for suicide by charcoal burning and from 11 March 2008 to 13 March 2010 for suicide by hydrogen sulfide), there were several events that aided in the promotion of media suicide reports that followed the recommendations for the media coverage of suicide; these included the enactment of the suicide prevention law in 2006 [34], emergency appeals by various organizations for regulations about media suicide reports [23,24,25], and an experience of a new type of suicide followed by increased number of suicide cases (i.e., a new type of suicide—excessive media coverage of a case in an inappropriate manner—suggested suicides) [18,34].

It is possible that the increased quality of the newspaper articles was due to the aforementioned factors [35]. Again, as a practical implication of the findings, it may be necessary to educate media professionals about not reporting suicide methods and about reporting the poor condition of the suicide survivor. In particular, the comparisons of newspaper articles about suicide prior to with those after the emergency recommendations issued during the hydrogen sulfide suicide epidemic in 2008 revealed that the proportions of articles not reporting a suicide note and address increased after the appeal (Table 5). Thus, the appeals for emergency recommendations may have influenced the newspaper suicide reports to follow the recommendations for media suicide coverage.

This study has several limitations that should be noted. First, since charcoal burning suicide reports after 11 March 2008, were not collected in the study, the periods of media coverage of the two types of suicide did not overlap. Thus, it is unclear whether the difference in reporting observed was due to a difference in the event difference (charcoal burning suicide vs. hydrogen sulfide suicide) or a difference in the time period (see mean violation score by year in Appendix). Second, the analyzed data were derived from a database that included newspaper articles without pictures. Thus, although the recommendations for media coverage about suicides suggest that a picture of a suicide is an important element to consider, the importance of pictures in the assessed articles could not be evaluated. Third, although the recommendations for media coverage about suicides indicate that a sensational article or the glorification of a suicide is another important element to consider, these items were not evaluated due to the difficulties associated with doing so. Fourth, media reports about suicide that were broadcast on television or published on the Internet were not evaluated in the present study. Additionally, although there were newspaper articles about suicides using methods other than charcoal burning or hydrogen sulfide during the study period, these articles were not included in the analyses. 

## 5. Conclusions

In conclusion, the present findings revealed the following: (1) the mean violation scores were 3.06 (±0.7) for all articles, 3.2 (±0.8) for articles about suicide by charcoal burning, and 2.9 (±0.7) for articles about suicide by hydrogen sulfide; (2) there was a significant difference between the violation scores for articles about suicide by hydrogen sulfide and those about suicide by charcoal burning; (3) the recommendations that were very likely to be violated included not reporting the suicide method, reporting information about where to seek help, and reporting the poor condition of a suicide survivor; and (4) the quality of newspaper articles improved, except for recommendations to not report the suicide method and to report the poor condition of a suicide survivor. These findings may be useful for the prevention of suggested suicides associated with media coverage.

## Figures and Tables

**Table 1:** Variables related to newspaper suicide articles which are used in a study (*n* = 4007).

Variables	Charcoal Burning (*n* = 2119)		Hydrogen Sulfide (*n* = 1888)		*p*
1. Months (%)					
January–March	673	(31.8)	137	(7.3)	<0.001
April–June	455	(21.5)	1453	(77.0)	
July–Sepetember	232	(11.0)	197	(10.4)	
October–December	759	(35.8)	101	(5.4)	
2. Day of the week (%)					
Sunday	238	(11.2)	209	(11.1)	<0.001
Monday	337	(15.9)	209	(11.1)	
Tuesday	381	(18.0)	270	(14.3)	
Wednesday	402	(19.0)	260	(13.8)	
Thursday	293	(13.8)	293	(15.5)	
Friday	240	(11.3)	336	(17.8)	
Saturday	228	(10.8)	311	(16.5)	
3. Newspaper companies (%)					
Asahi	301	(14.2)	202	(10.7)	<0.001
Nihonkeizai	78	(3.7)	37	(2.0)	
Mainichi	434	(20.5)	444	(23.5)	
Yomiuri	391	(18.5)	308	(16.3)	
Sannkei	198	(9.3)	118	(6.3)	
Others	717	(33.8)	779	(41.3)	
4. Words count, mean (±SD)	624.2	(580.2)	543.7	(652.7)	<0.001
5. Front page article (front page)	78	(3.7)	34	(1.8)	<0.001
6. Reporting suicide method (yes), n (%)	2002	(94.5)	1872	(99.2)	<0.001
7. Reporting death note (yes), n (%)	577	(27.2)	321	(17.0)	<0.001
8. Reporting the reason for suicide (yes), n (%)	228	(10.8)	83	(4.4)	<0.001
9. Reporting the information about where to seek help (no), n (%)	2077	(98.0)	1472	(78.0)	<0.001
10. Reporting the poor condition of a suicide survivor (no), n (%)	1807	(85.3)	1770	(93.8)	<0.001
11. Violation score, mean (±SD)	3.2	(0.8)	2.9	(0.7)	<0.001
12. Reporting age of a suicide (yes), n (%)	1646	(77.7)	1480	(78.4)	0.587
13. Reporting gender of a suicide (yes), n (%)	2063	(97.4)	1723	(91.3)	<0.001
14. Reporting profession of a suicide (yes), n (%)	1035	(48.8)	990	(52.4)	<0.001
15. Reporting address of a suicide (yes), n (%)	1365	(64.4)	1393	(73.8)	0.023
16. Reporting site of suicide (yes), n (%)	1537	(72.5)	1743	(92.3)	<0.001

n: Number of articles; SD: Standard deviation.

**Table 2:** Number of newspaper articles about suicide according to violation score and type of suicide.

Violation Score Groups	Charcoal Burning (*n* = 2119)		Hydrogen Sulfide (*n* = 1888)		*p*
0–2	317	(15.0)	480	(25.4)	<0.001
3–4	1687	(79.6)	1360	(72.0)	
5–6	115	(5.4)	48	(2.5)	

**Table 3:** Number of newspaper articles about suicide according to type of suicide and violation score.

Items of the Recommendation for Suicide Articles	0–2					3–4					5–6				
	Charcoal Burning (*n* = 317)		Hydrogen Sulfide (*n* = 480)		*p*	Charcoal Burning (*n* = 1687)		Hydrogen Sulfide (*n* = 1360)		*p*	Charcoal Burning (*n* = 115)		Hydrogen Sulfide (*n* = 48)		*p*
1. Front page article (yes), n (%)	1	(0.3)	2	(0.4)	0.819	67	(4.0)	30	(2.2)	0.006	10	(8.7)	2	(4.2)	0.313
2. Reporting suicide method (yes), n (%)	217	(68.5)	468	(97.5)	<0.001	1670	(99.0)	1356	(99.7)	0.018	115	(100.0)	48	(100.0)	
3. Reporting death note (yes), n (%)	1	(0.3)	1	(0.2)	0.767	461	(27.3)	272	(20.0)	<0.001	115	(100.0)	48	(100.0)	
4. Reporting reason for suicide (yes), n (%)	4	(1.3)	5	(1.0)	0.773	116	(6.9)	32	(2.4)	<0.001	108	(93.9)	46	(95.8)	0.625
5. Reporting information of where to seek help (no), n (%)	286	(90.2)	86	(17.9)	<0.001	1676	(99.4)	1338	(98.4)	0.010	115	(100.0)	48	(100.0)	
6. Reporting poor condition of a suicide survivor (no), n (%)	95	(30.0)	377	(78.5)	<0.001	1597	(94.7)	1345	(98.9)	<0.001	115	(100.0)	48	(100.0)	
7. Reporting age of a suicide (yes), n (%)	213	(67.2)	286	(59.6)	0.030	1323	(78.4)	1146	(84.3)	<0.001	110	(95.7)	48	(100.0)	0.142
8. Reporting gender of a suicide (yes), n (%)	304	(95.9)	385	(80.2)	<0.001	1648	(97.7)	1291	(94.9)	<0.001	111	(96.5)	47	(97.9)	0.638
9. Reporting profession of a suicide (yes), n (%)	132	(41.6)	185	(38.5)	0.382	825	(48.9)	761	(56.0)	<0.001	78	(67.8)	44	(91.7)	0.001
10. Reporting address of a suicide (yes), n (%)	196	(61.8)	286	(59.6)	0.526	1075	(63.7)	1065	(78.3)	<0.001	94	(81.7)	42	(87.5)	0.367
11. Reporting site of suicide (yes), n (%)	175	(55.2)	396	(82.5)	<0.001	1280	(75.9)	1300	(95.6)	<0.001	82	(71.3)	47	(97.9)	<0.001

Note: Violation score is a total of “yes” or “no” to Nos. 1–6 items in the table. n: Number of articles.

**Table 4:** Logistic regression analyses for hydrogen-sulfide suicide (vs. charcoal-burning suicide) and items that should be avoided when reporting suicides.

Model	Front Page Article (Yes)			Reporting Suicide Method (Yes)			Reporting Suicide Note (Yes)		
	OR	95% CI	*p*	OR	95% CI	*p*	OR	95% CI	*p*
Unadjusted	0.48	(0.32, 0.72)	<0.001	6.84	(4.04, 11.57)	<0.001	0.55	(0.47, 0.64)	<0.001
Adjusted for words count	0.49	(0.32, 0.73)	0.001	7.36	(4.21, 12.86)	<0.001	0.51	(0.44, 0.60)	<0.001
Adjusted for words count and newspaper companies	0.45	(0.30, 0.68)	<0.001	7.58	(4.32, 13.31)	<0.001	0.53	(0.45, 0.62)	<0.001
Adjusted for words count, newspaper companies, month, and day of the week	0.35	(0.21, 0.58)	<0.001	5.10	(2.62, 9.91)	<0.001	0.55	(0.46, 0.67)	<0.001
Hosmer-Lemeshow test (full model)			0.124			0.385			0.404
**Model**	**Reporting Reason for Suicide (Yes)**			**Reporting Information of Where to Seek Help (No)**			**Reporting Poor Condition of a Suicide Survivor (No)**		
	**OR**	**95% CI**	***p***	**OR**	**95% CI**	***p***	**OR**	**95% CI**	***p***
Unadjusted	0.38	(0.29, 0.49)	<0.001	0.07	(0.05, 0.10)	<0.001	2.59	(2.07, 3.23)	<0.001
Adjusted for words count	0.39	(0.30, 0.50)	<0.001	0.05	(0.03, 0.07)	<0.001	2.56	(2.05, 3.20)	<0.001
Adjusted for words count and newspaper companies	0.41	(0.31, 0.53)	<0.001	0.04	(0.03, 0.06)	<0.001	2.52	(2.02, 3.15)	<0.001
Adjusted for words count, newspaper companies, month, and day of the week	0.36	(0.27, 0.49)	<0.001	0.14	(0.10, 0.21)	<0.001	2.65	(2.03, 3.46)	<0.001
Hosmer-Lemeshow test (full model)			0.767			<0.001			0.299

OR, odds ratio; CI, confidence interval.

**Table 5:** Proportions of newspaper articles about suicide according to period of article publication.

Items of the Recommendation for Suicide Articles	Pre Appeal (*n* = 244)		Post Appeal (*n* = 1603)		*p*
1. Front page article (yes), % (n)	0.8	(2)	0.032	(32)	0.203
2. Reporting suicide method (yes), % (n)	100.0	(244)	99.0	(1587)	0.117
3. Reporting death note (yes), % (n)	23.0	(56)	15.7	(252)	0.005
4. Reporting reason for suicide (yes), % (n)	1.2	(3)	4.7	(75)	0.013
5. Reporting information of where to seek help (no), % (n)	73.8	(180)	78.0	(1251)	0.137
6. Reporting poor condition of a suicide survivor (no), % (n)	96.3	(235)	93.3	(1495)	0.069
7. Reporting age of a suicide (yes), % (n)	79.9	(195)	78.0	(1250)	0.494
8. Reporting gender of a suicide (yes), % (n)	91.0	(222)	91.1	(1461)	0.936
9. Reporting profession of a suicide (yes), % (n)	53.7	(131)	52.1	(835)	0.641
10. Reporting address of a suicide (yes), % (n)	81.2	(198)	72.4	(1161)	0.004
11. Reporting site of suicide (yes), % (n)	94.7	(231)	91.8	(1471)	0.116

Note: Pre appeal: 11 March 2008–17 April 2008; Post appeal: 18 April 2008–13 March 2010; n: Number of articles.

## References

[1] Stack S. (2000). Media impacts on suicide: A quantitative review of 293 findings. Soc. Sci. Q..

[2] Pirkis J.E., Burgess P.M., Francis C., Blood R.W., Jolley D.J. (2006). The relationship between media reporting of suicide and actual suicide in Australia. Soc. Sci. Med..

[3] World Health Organization (WHO) (2008). Preventing Suicide: A Resource for Media Professionals.

[4] Niederkrotenthaler T., Voracek M., Herberth A., Till B., Strauss M., Etzersdorfer E., Eisenwort B., Sonneck G. (2010). Role of media reports in completed and prevented suicide: Werther v. Papageno effects. Br. J. Psychiatry.

[5] Niederkrotenthaler T., Fu K., Yip P.S.F., Fong D.Y.T., Stack S., Cheng Q., Pirkis J. (2012). Changes in suicide rates following media reports on celebrity suicide: A meta-analysis. J. Epidemiol. Community Health.

[6] Sisask M., Värnik A. (2012). Media roles in suicide prevention: A systematic review. Int. J. Environ. Res. Public Health.

[7] Blumenthal S., Bergner L. (1973). Suicide and newspapers: A replicated study. Am. J. Psychiatry.

[8] Gould M.S., Shaffer D. (1986). The impact of suicide in television movies: Evidence of imitation. N. Engl. J. Med..

[9] Lee A., Ahn M.H., Lee T.Y., Park S., Hong J.P. (2014). Rapid spread of suicide by charcoal burning from 2007 to 2011 in Korea. Psychiatry Res..

[10] Hagihara A., Tarumi K., Abe T. (2007). Media suicide-reports, Internet use and the occurrence of suicides between 1987 and 2005 in Japan. BMC Public Health.

[11] Nakamura M., Yasunaga H., Toda A.A., Sugihara T., Imamura T. (2012). The impact of media reports on the 2008 outbreak of hydrogen sulfide suicides in Japan. Int. J. Psychiatry Med..

[12] Stack S. (2003). Media coverage as a risk factor in suicide. J. Epidemiol. Community Health.

[13] Stack S. (1987). Celebrities and suicide: A taxonomy and analysis, 1948–1983. Am. Sociol. Rev..

[14] Wasserman I.M. (1984). Imitation and suicide: A reexamination of the Werther effect. Am. Sociol. Rev..

[15] The Suicide Prevention Resource Center (SPRC) Recommendations for Reporting Suicide. http://reportingonsuicide.org/wp-content/themes/ros2015/assets/images/Recommendations-eng.pdf.

[16] Center for Disease Control and Prevention (SPRC) Suicide Contagion and the Reporting of Suicide: Recommendations from a National Workshop. http://www.cdc.gov/mmwr/preview/mmwrhtml/00031539.htm.

[17] Ueda S. Research of Actual Condition and Prevention of Group Suicide via the Internet. http://ikiru.ncnp.go.jp/report/ueda16t/ueda16t-1.pdf.

[18] Rajagopal S. (2004). Suicide pacts and the internet: Complete strangers may make cyberspace pacts. BMJ.

[19] Ogiwara A., Watanabe E. (2009). A study on suicide-reports on newspaper in Japan. Suicide Prev. Crisis Interv..

[20] The Office for Suicide Prevention, Cabinet Office of Japan Research for Suicide by Hydrogen Sulfide and Media Reports in 2008. http://www8.cao.go.jp/jisatsutaisaku/whitepaper/w-2009/html/honpen/part3/s3_1_01_sanko.html.

[21] Shinbun A. (2008). Three young people meeting for the first time via the Internet and committing suicide as a group using hydrogen sulfide. Asahi Shinbun Morning Edition.

[22] The Number of Suicides by Hydrogen Sulfide between January and November, 2008 Exceeds 1000. http://news.yahoo.co.jp/pickup/29810.

[23] Lifelink (NPO for Suicide Prevention) A Request to Improve Suicide Media Reports on *Ijime. Jisatsu.* (Suicide Caused by Bullying) on 30 October 2006. http://www.lifelink.or.jp/hp/jisatsuhoudou.html#Guideline.

[24] Cabinet Office of Japan Government Suicide Prevention: Guidelines for Media. http://www8.cao.go.jp/jisatsutaisaku/link/kanren.html.

[25] Japanese Association for Suicide Prevention A Request to Media Professionals to Prevent Imitative Suicide Using Hydrogen Sulfide. http://www8.cao.go.jp/jisatsutaisaku/h2s/.

[26] Au J.S.K., Yip P.S.F., Chan C.L.W., Law Y.W. (2004). Newspaper reporting of suicide cases in Hong Kong. Crisis.

[27] Ji N., Lee W., Noh M., Yip P. (2014). The impact of indiscriminate media coverage of a celebrity suicide on a society with a high suicide rate: Epidemiological findings on copycat suicides from South Korea. J. Affect. Disord..

[28] Cheng A.T.A., Hawton K., Lee C.T.C., Chen T.H.H. (2007). The influence of media reporting of the suicide of a celebrity on suicide rates: A population-based study. Int. J. Epidemiol..

[29] The Office for Suicide Prevention, Cabinet Office of Japan Announcement about the Measures for Prevention of Suicide by Hydrogen Sulfide. http://www8.cao.go.jp/jisatsutaisaku/suisin/k_3/pdf/s1.pdf.

[30] The Office for Suicide Prevention, Cabinet Office of Japan Reminder about Hydrogen Sulfide Gas Outbreak. http://www8.cao.go.jp/jisatsutaisaku/h2s/pdf/notice.pdf.

[31] World Health Organization (WHO) (2000). Preventing Suicide: A Resource for Media Professionals.

[32] Sakamoto S., Tanaka E., Kageyama T. (2006). The present state and problems in suicide reports in Japanese newspapers. A content analysis study of suicide reports after “internet suicide pacts”. Jpn. J. Ment. Health.

[33] Pirkis J., Burgess P., Blood R.W., Francis C. (2007). The newsworthiness of suicide. Suicide Life Threat. Behav..

[34] Basic Act on Suicide Prevention (Act. Law No. 85). http://www.mhlw.go.jp/file/06-Seisakujouhou-12200000-Shakaiengokyokushougaihokenfukushibu/0000122062.pdf.

[35] Pirkis J., Dare A., Blood R.W., Rankin B., Williamson M., Burgess P., Jolley D. (2009). Changes in media reporting of suicide in Australia between 2000/01 and 2006/07. Crisis.

