# Correction: Nabeshima, Y., et al. Analysis of Japanese Articles about Suicides Involving Charcoal Burning or Hydrogen Sulfide Gas. *Int. J. Environ. Res. Public Health* 2016, *13*, 1013

**DOI:** 10.3390/ijerph14121479

**Published:** 2017-12-01

**Authors:** Yoshihiro Nabeshima, Daisuke Onozuka, Takanari Kitazono, Akihito Hagihara

**Affiliations:** 1 Department of Medicine and Clinical Science, Graduate School of Medical Sciences, Kyushu University, 3-1-1 Maidashi, Higashi-ku, Fukuoka 812-8582, Japan; kendai.nabe@gmail.com (Y.N.); kitazono@intmed2.med.kyushu-u.ac.jp (T.K.); 2 Department of Health Communication, Graduate School of Medical Sciences, Kyushu University, 3-1-1 Maidashi, Higashi-ku, Fukuoka 812-8582, Japan; onozukad@hcam.med.kyushu-u.ac.jp

We wish to make the following three corrections to the published paper [1]. First, one of the objectives of this study was incorrect. The correct objective was to determine whether the quality of newspaper articles on suicide that were published following the appeals for emergency recommendation for suicide reporting by the media showed any improvement of the guideline adherence compared with those published before the appeals. Second, the period during which there was a significant difference between the charcoal-burning and hydrogen-sulfide groups (*p* < 0.001)—approximately 70% of the articles reported were about charcoal-burning suicides—was incorrect. The correct period was the January–September period. Third, the statement that there was a higher proportion of articles about suicide by charcoal burning than about suicide by hydrogen sulfide violating the elements of violation score was incorrect. The correct statement is that there was a higher proportion of articles about suicide by hydrogen sulfide than about suicide by charcoal burning following the implementation of guidelines on reporting suicides in the media; the exceptions concerned reporting the suicide method and not reporting the poor condition of the suicide survivor.

We apologize for any inconvenience caused to the readers by this error. The article will be updated and the original will remain on the article webpage.
